# Differences in Homomorphic Sex Chromosomes Are Associated with Population Divergence in Sex Determination in *Carinascincus ocellatus* (Scincidae: Lygosominae)

**DOI:** 10.3390/cells10020291

**Published:** 2021-02-01

**Authors:** Peta Hill, Foyez Shams, Christopher P. Burridge, Erik Wapstra, Tariq Ezaz

**Affiliations:** 1 Discipline of Biological Sciences, University of Tasmania, Private Bag 5, Sandy Bay, TAS 7000, Australia; Chris.Burridge@utas.edu.au (C.P.B.); Erik.Wapstra@utas.edu.au (E.W.); 2 Institute for Applied Ecology, University of Canberra, Bruce, ACT 2601, Australia; Foyez.Shams@canberra.edu.au (F.S.); Tariq.Ezaz@canberra.edu.au (T.E.)

**Keywords:** cryptic sex chromosomes, karyotype, GSD, TSD, *Niveoscincus*

## Abstract

Sex determination directs development as male or female in sexually reproducing organisms. Evolutionary transitions in sex determination have occurred frequently, suggesting simple mechanisms behind the transitions, yet their detail remains elusive. Here we explore the links between mechanisms of transitions in sex determination and sex chromosome evolution at both recent and deeper temporal scales (<1 Myr; ~79 Myr). We studied a rare example of a species with intraspecific variation in sex determination, *Carinascincus ocellatus*, and a relative, *Liopholis whitii*, using c-banding and mapping of repeat motifs and a custom Y chromosome probe set to identify the sex chromosomes. We identified both unique and conserved regions of the Y chromosome among *C. ocellatus* populations differing in sex determination. There was no evidence for homology of sex chromosomes between *C. ocellatus* and *L. whitii*, suggesting independent evolutionary origins. We discuss sex chromosome homology between members of the subfamily Lygosominae and propose links between sex chromosome evolution, sex determination transitions, and karyotype evolution.

## 1. Introduction

Development as male or female is central to sexual reproduction [1]. Sex determination decides the sexual fate of the developing gonad, and because the outcome is highly conserved, the underlying mechanism is also expected to be conserved [2]. However, sex determination is surprisingly labile in vertebrates, which has generated considerable scientific interest [1,3,4,5,6,7]. The diversity in sex determination in vertebrates is also accompanied by morphological and genetic diversity in sex chromosomes [5,8,9,10,11]. Because sex chromosomes also play a central role in postzygotic isolation and speciation [12,13,14,15], they may simultaneously reinforce any ongoing divergence in sex determination, raising fundamental questions about the links between sex chromosome evolution, sex determination transitions, and lineage divergence [16,17].

Our understanding of the potential contribution of sex chromosomes to transitions in sex determination relies on basic knowledge of sex chromosome evolution. The classic theory of sex chromosome evolution describes how they first arise when recombination around a sex determining locus is suppressed [1,11]. Recombination suppression on sex chromosomes is marked by the accumulation of inversions [18], heterochromatinisation [19], transposable elements and other repetitive sequences such as microsatellite motifs [20], but it is unknown whether this is a cause or consequence of recombination suppression [21]. The evolutionary trajectory of sex chromosomes in all but a few bird and mammal species has resulted in sex chromosomes progressing from homomorphy to heteromorphy [22,23,24] and the origin of sex chromosomes in these lineages is ancient (approximately 140 and 180 mya ago in birds and mammals, respectively [1,25]. In contrast, sex chromosomes are diverse in plants, invertebrates, fish and reptiles and several mechanisms contribute to this diversity [26,27,28,29,30]. For example, a pair of autosomes may usurp existing sex chromosomes via the acquisition of a new sex determining locus, or sex chromosome-autosome fusions may result in a new sex chromosome system [31]. Further, thermosensitivity in sex determination can lead to transitions between alternative systems of heterogamety (XY male, ZW female) or from genetic to environmental sex determination, where sex chromosomes are lost [32,33]. Sex chromosome evolution therefore follows diverse pathways, the dynamics of which are poorly understood.

Lizards provide an opportunity to understand sex chromosome evolution, and possible links to transitions in sex determination and speciation. However, the mechanisms that underpin transitions in sex determination and how they relate to sex chromosomes and speciation remain unclear because information regarding sex determination and sex chromosomes is lacking for the vast majority of lineages [1,34]. Among lizards, despite evidence of homology of sex-linked chromosomal regions among some species [35,36,37,38,39], sex chromosomes display extraordinary variation in morphology. Within the family Scincidae, XY heterogamety is prevalent, however, the recent discovery of a skink with ZW heterogamety [40] highlights at least one transition between these systems. In addition, heteromorphic and homomorphic sex chromosomes are broadly distributed throughout skinks [41,42,43] and skink species with sex chromosomes also exhibit temperature sensitivity of sex determination [35,44,45,46,47,48,49]. Sex determination transitions and sex chromosome evolution have occurred frequently in this group and the diploid chromosome complement is varied [43].

*Carinascincus ocellatus* (Scincidae) is a rare example of a species exhibiting population divergence in sex determination; a high elevation population (1200 masl) has genetic sex determination (GSD) with XY heterogamety while a low elevation population (50 masl) has thermosensitive XY GSD [48,50,51,52]. Herein, we describe the low elevation population as having GSD+EE (Environmental Effects; *sensu* Valenzuela, et al. [53]). Shared sex-linked markers in these populations define conserved sex chromosome sequence. However, these populations differ in the linkage disequilibrium among these shared sex-linked markers, and they also possess population-specific sex-linked markers, suggesting sex chromosome divergence [48]. Recombination is more disrupted in the high elevation GSD population, representing molecular evidence of more progressed sex chromosome evolution. However, a karyotype analysis did not reveal differentiated sex chromosomes in *C. ocellatus* [54].

**Figure 1:**
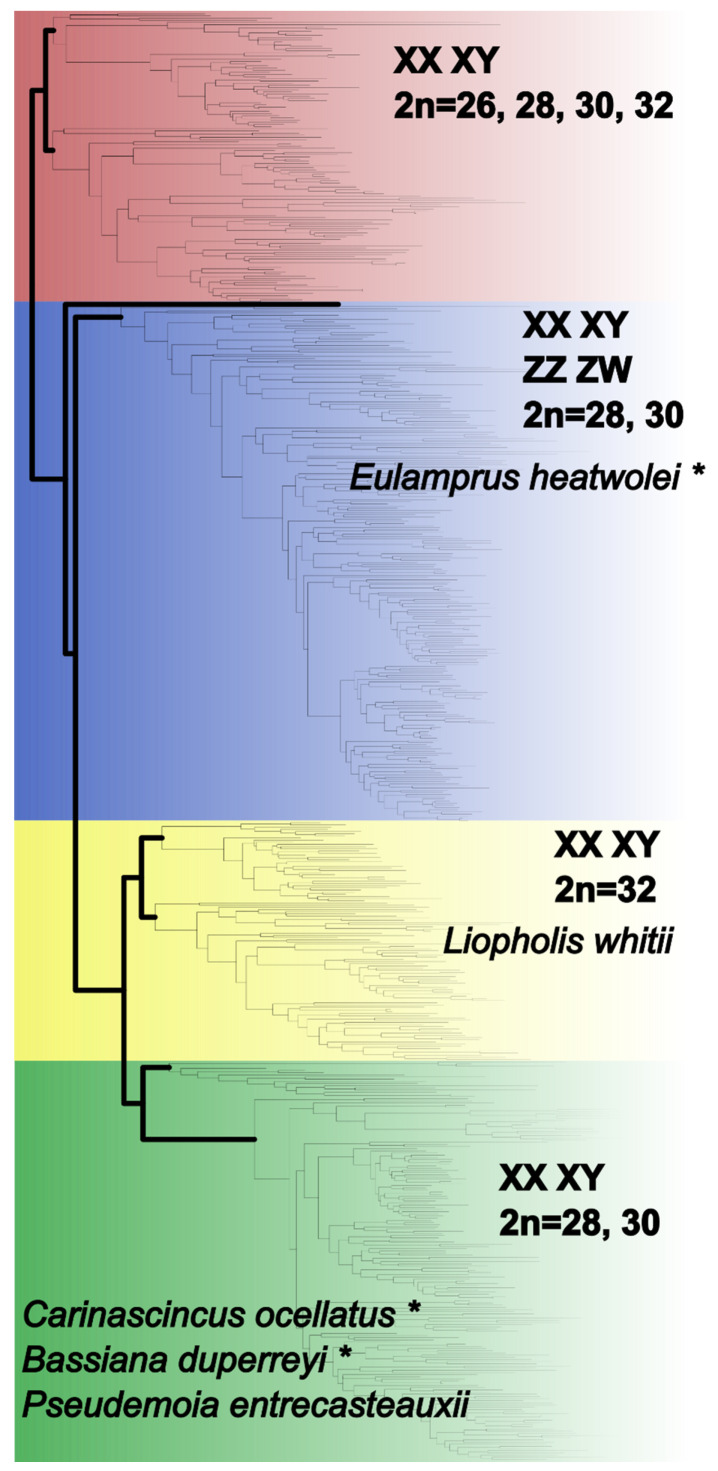
Variation in diploid chromosome complement and sex determination in Scincid subfamilies Scincinae (red) and Lygosominae (blue, yellow and green). The clades to which our study species (*Carinascincus ocellatus* and *Liopholis whitii*) belong and other species of interest discussed herein are included. The age of the split between clades containing *C. ocellatus*, *L. whitii* and *E. heatwolei* is estimated as 79 mya [56]. Adapted from Pyron, et al. [59] and Olmo and Signorino [43]. * denotes species where thermosensitive sex determination has been reported [35,45,46,49].

Here we examine the karyotype of *C. ocellatus* (2n = 30) and compare it to that of *Liopholis whitii* (2n = 32, sex chromosomes homomorphic [41]) from the subfamily Lygosominae (Figure 1), to identify sex chromosomes. Specifically, we examined the high and low elevation populations of *C. ocellatus*, which diverged less than 1 Mya [55], to test whether intraspecific divergence in sex determination is reflected by gross sex chromosome variation. In addition, we provide a phylogenetic assessment of sex chromosome evolution through comparison between *C. ocellatus* and *L. whitii*, which diverged approximately 79 Mya (Figure 1, divergence data retrieved from TimeTree, [56]), to test for homology of sex chromosomes in these lineages and to understand the mechanisms of sex chromosome evolution across deeper temporal scales. We examined metaphase spreads of males and females and used standard c-banding plus a custom probe set designed from *C. ocellatus* Y-linked sequence. In addition, we mapped repeats (AGAT and telomere) to the karyotypes of both species because of their association with sex chromosomes in a broad range of reptiles [57,58]. We discuss sex chromosome evolution in the context of sex determination transitions and karyotype evolution.

## 2. Materials and Methods

### 2.1. Study Species

**Figure 2:**
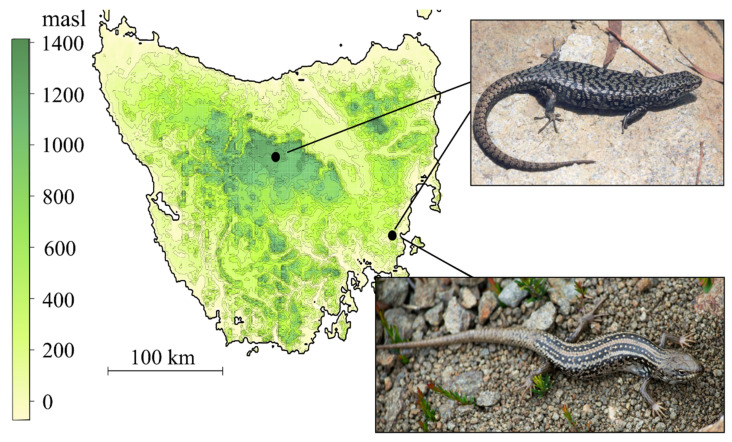
Sampling locations of *Carinascincus ocellatus* (inset, upper) and *Liopholis whitii* (inset, lower). *C. ocellatus* were sampled from high and low elevation populations exhibiting GSD and GSD+EE, respectively.

*Carinascincus ocellatus* is a small (60 to 80 mm snout-vent length, 3 to 10 g) viviparous skink endemic to Tasmania, with a broad altitudinal distribution from sea level to 1200 m [60]. We collected three males and three females from populations representing the climatic extremes of this species’ range: a cool temperate low elevation population with GSD+EE (42°34′ S, 147°52′ E; elevation 50 m, Table 1, Figure 2) and a high elevation, cold temperate population with GSD (41°51′ S, 146°34′ E; elevation 1200 m, Table 1, Figure 2). Long term data on these populations consistently documents their divergent sex determination [50,51,52].

*Liopholis whitii* is a medium viviparous skink (snout-vent length < 100 mm) found throughout south eastern Australia. We examined three males and three females from the same low elevation location as *C. ocellatus* (Table 1, Figure 2). All guidelines and procedures for the use of animals were approved by the University of Tasmania animal ethics committee (no. A0017006).

### 2.2. Blood Culture and Metaphase Chromosome Preparations

Metaphase spreads were obtained from whole blood using a modified version of the protocol described in Ezaz, et al. [61]. Briefly, 50 µL of whole blood was taken via heparinised capillary tube from the sub-orbital sinus to set up 2 mL cultures in Dulbecco’s modified Eagle’s medium (DMEM; Gibco, Thermo Fisher Pty Ltd., Scoresby Victoria, Australia) supplemented with 20% fetal bovine serum (Gibco), 4% antibiotic-antimycotic solution (Gibco) and 8% phytohemagglutinin from *Phaseolus vulgaris* (PHA-M, Sigma-Aldrich, St. Louis, MI, USA). Cells were cultured for 3 days at 28 °C and 5% CO_2_. Cell division was arrested 3.5 h prior to harvesting, using 0.05 μg/mL colcemid (Roche, Mannheim, Germany). Cells were treated with hypotonic solution (75 mM KCl) at 37 °C and fixed in 3:1 methanol-acetic acid. The cell suspension was dropped onto glass slides, dehydrated through an ethanol series of 70%, 90%, 100%, air dried and stored at −80 °C.

### 2.3. Development of Sex-Linked Probe Set for Chromosome Mapping

Thirty-two loci with sex-linked genotypes from double-digest, restriction-site associated DNA sequencing (RAD-seq) of *C. ocellatus* [48,62] were chosen for inclusion in our custom probe set design (Table S1). Of these, 26 represent Y-linked sequence (only present in males) common to both populations. Six loci were on both X and Y chromosomes, but with single nucleotide polymorphisms (SNPs) segregating chromosomes (males heterozygous, females homozygous). Oligonucleotides were synthesized from these Y-linked sequences and SNP RAD-tag sequences (38–69 bp) and each fragment was end-labelled with 3X ATTO594 dye (Arbor Biosciences; https://arborbiosci.com).

### 2.4. C-Banding

We analysed 1–2 males and females from *L. whitii* and each *C. ocellatus* population and examined 3–31 cells per individual (Table 1). C-banding was performed as described by Sumner [63], Ezaz, et al. [61] and Shams, et al. [64] with slight modification. Briefly, 10–15 µL of cell suspension was dropped on a glass slide, air dried and aged for 60 min on a 60 °C hot plate. Aged slides were treated with 0.2 N HCl at room temperature for 30 min, then rinsed in distilled water and subsequently treated with 5% Ba(OH)_2_ at 50 °C for 6 min. Slides were again rinsed in distilled water and then incubated at 60 °C in 2× SSC (saline sodium citrate) for 1 h. Finally, for DAPI (4′,6-diamidino-2-phenylindole) staining, the slides were mounted with antifade medium Vectashield (Vector Laboratories, Burlingame, CA, USA) containing 1.5 mg/mL DAPI.

### 2.5. Fluorescence In Situ Hybridisation (FISH) with Microsatellite Motif, Telomere and Y-Linked Probe Set

We analysed up to three males and three females from *L. whitii* and each *C. ocellatus* population and examined 15–43 cells per individual (Table 1) to determine distributions of the (AGAT)_8_ microsatellite motif, (TTAGGG)_7_ telomeric repeats and the custom Y-linked probe set in males and females. The microsatellite motif (AGAT)_8_ was chosen because of its association with sex chromosomes in multiple reptilian groups including the Y chromosome in a closely related skink, *Bassiana duperreyi* (Figure 1, [57]). Telomere probe and microsatellite motif probes were Cy3-labelled (GeneWorks, Hindmarsh, South Australia, Australia). FISH was performed as described in Ezaz, et al. [61] and Matsubara, et al. [65] with slight modifications. Briefly, 500 ng of microsatellite and telomere oligonucleotides and 135 ng of Y-linked probe set were added to 12.5 µL hybridisation buffer (50% formamide, 10% dextran sulphate, 2× SSC, 40 mM sodium phosphate pH 7.0 and 1× Denhardt’s solution) and warmed to 37 °C. The hybridisation mixture was placed onto the slide and sealed with a coverslip and rubber cement. Probe and chromosome DNA were denatured at 68.5 °C for 5 min followed by incubation in a moist hybridisation chamber at 37 °C for 24–48 h. Slides were then washed in 0.4× SSC, 0.3% IGEPAL (Sigma) at 60 °C for 2 min followed by 2× SSC, 0.1% IGEPAL at room temperature for 1 min. Slides were dehydrated by ethanol series and air dried. Finally, the slides were mounted with antifade medium Vectashield containing 1.5 mg/mL DAPI.

In addition to the individual FISH experiments, we performed sequential FISH initially with the custom Y-linked probe set followed by the (AGAT)_8_ probe on male metaphase from both *C. ocellatus* populations. Hybridisation signals from the Y-linked probe set were photographed, metaphase positions recorded then slides were washed in 0.4x SSC at 60 °C for 1 min, followed by 2× SSC at room temperature for 2 min before hybridisation with the (AGAT)_8_ probe. Because both probes were labelled using fluorophores with similar excitation-emission wavelengths, it was not possible to resolve these signals using our existing microscope filter systems. Therefore, photos were merged in Photoshop (CS6) with the colour of the Y-linked signal altered digitally so its association with repeat motifs could be examined.

### 2.6. Microscopy and Image Analysis

Chromosome images were captured using a Zeiss Axio Scope A1 epifluorescence microscope fitted with a high-resolution microscopy camera AxioCam MRm Rev. 3 (Carl Zeiss Ltd. Oberkochen, Germany) and a Leica DM6 B (Leica microsystems, Macquarie park, Australia). Images were analysed using Metasystems Isis FISH Imaging System V 5.5.10 software (Metasystems, Altlussheim, Germany) as well as Thunder Imager 3D (Leica microsystems).

### 2.7. Marker Homology

We used NCBI BLAST [66] to identify homologs of our markers with publicly available sequences from vertebrates. Specifically, we searched the “nr” database with the default settings in NCBI’s blastn suite and report matches related to sex determination or sexual development with e-values < 0.001.

## 3. Results

### 3.1. DAPI Karyotypes

**Figure 3:**
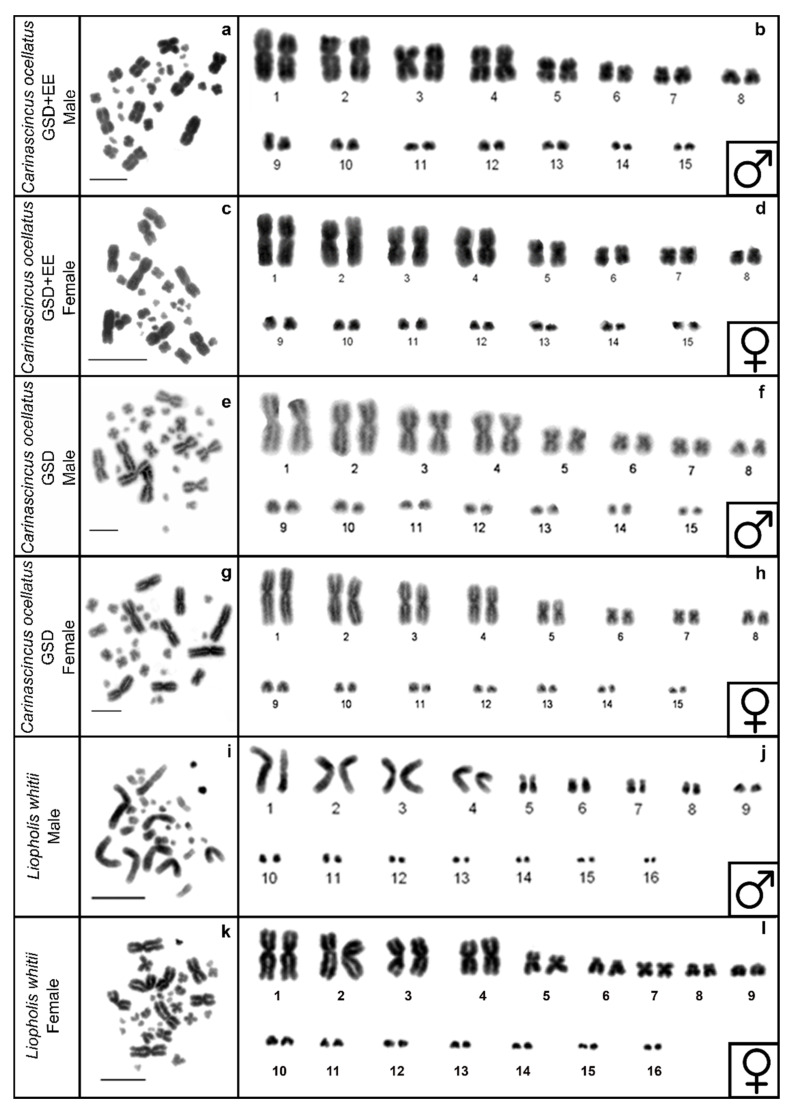
DAPI stained (inverted) metaphase spread and karyotypes in GSD+EE (low elevation) *Carinascincus ocellatus* male (**a**,**b**) and female (**c**,**d**), GSD (high elevation) *C. ocellatus* male (**e**,**f**) and female (**g**,**h**) and *Liopholis whitii* male (**i**,**j**) and female (**k**,**l**). Scale bar represents 10 µm.

The karyotype of GSD and GSD+EE populations of *C. ocellatus* is 2n = 30, represented by eight pairs of macrochromosomes and seven pairs of microchromosomes (Figure 3a–h), while the karyotype of *L. whitii* is 2n = 32, represented by nine pairs of macrochromosomes and seven pairs of microchromosomes (Figure 3i-l). These are consistent with karyotypes described in Donnellan [54] and Donnellan [41]. Sex chromosomes are homomorphic (Figure 3a–l).

### 3.2. Custom Y-Linked C. ocellatus Probe Set

**Figure 4:**
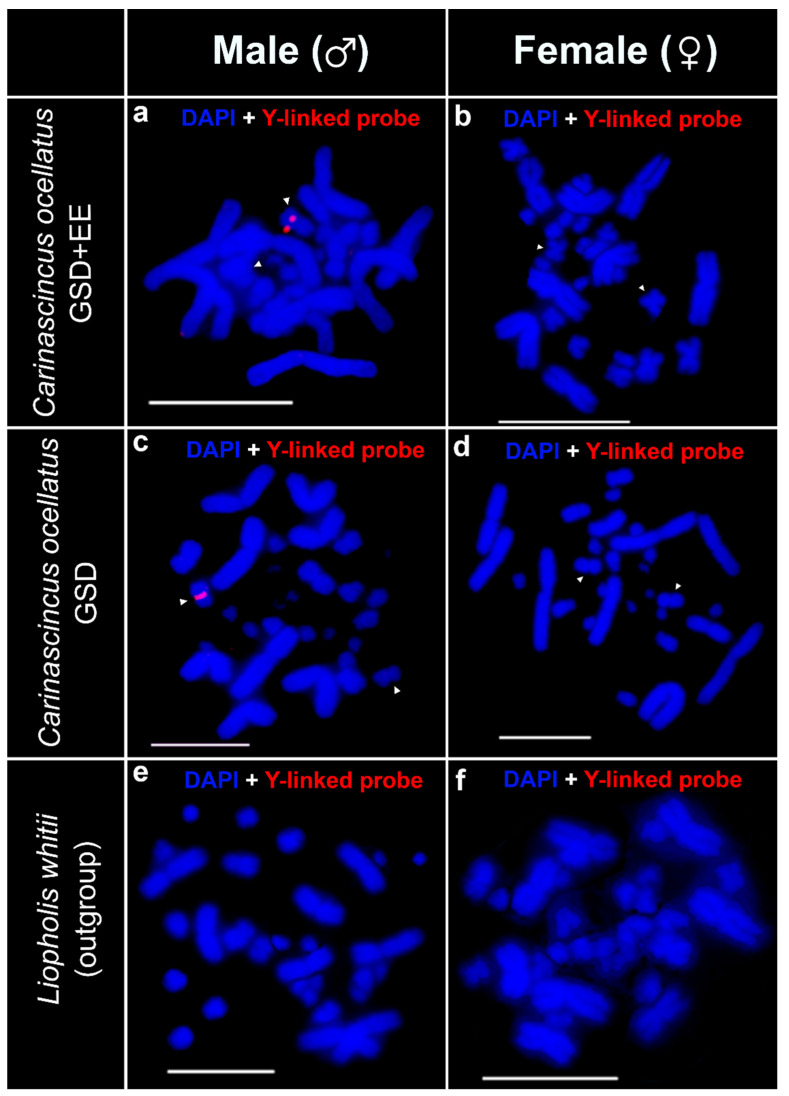
Chromosomal locations of *Carinascincus ocellatus* Y-linked FISH probe set (red) in GSD+EE (low elevation) *C. ocellatus* male (**a**) and female (**b**), GSD (high elevation) *C. ocellatus* male (**c**) and female (**d**), and *Liopholis whitii* male (**e**) and female (**f**). White arrowhead indicates X and Y (the homologous pair) chromosomes in *C. ocellatus*. Scale bar represents 10 µm.

Our Y-linked probe set hybridised adjacent to the centromere on a single chromosome in males of both populations of *C. ocellatus*, and therefore identified the Y chromosome in both populations (Figure 4a,c). We did not detect any signal on females of either population, evidence that this probe set is specific to Y-linked sequences and does not contain sequences that are present on the X chromosome in quantities large enough to detect with FISH (Figure 4b,d). We did not detect any hybridisation signals on *L. whitii* (Figure 4e,f).

### 3.3. C-Banding

**Figure 5:**
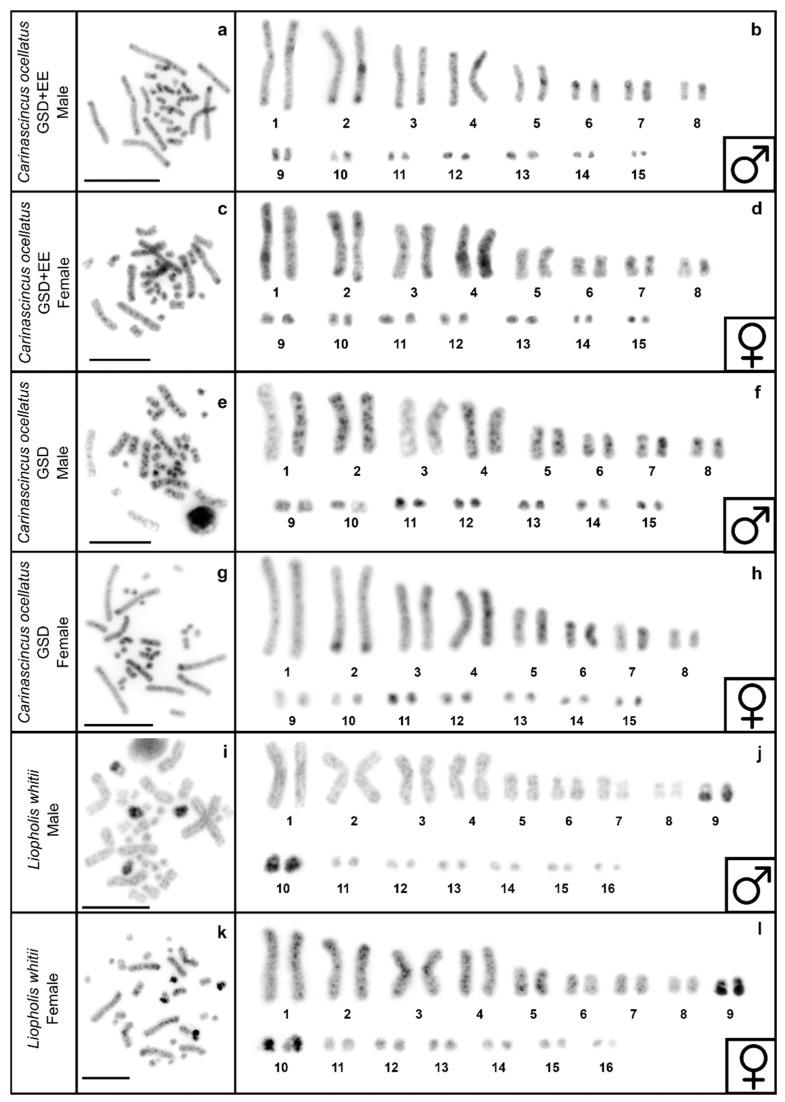
C-banded spread and karyotypes in GSD+EE (low elevation) *Carinascincus ocellatus* male (**a,b**) and female (**c,d**), GSD (high elevation) *C. ocellatus* male (**e,f**) and female (**g,h**) and *Liopholis whitii* male (**i,j**) and female (**k,l**). Scale bar represents 10 µm.

Accumulation of multiple heterochromatic bands are consistently observed across all macrochromosomes in both populations of *C. ocellatus*, while only one major band was observed in all microchromosomes. At least four microchromosomes are highly heterochromatic (Figure 5a–h). Comparisons of C-banded karyotypes between males and females of both populations of *C. ocellatus* identified one of the homologs of chromosome pair 7 as highly heterochromatic in males but not females from the GSD (high elevation) population (Figure 5e–h); no sex specific heterochromatinisation was observed at low elevation (Figure 5a–d). This corroborates the signal from the Y-specific probe and confirms the Y chromosome has a region of heterochromatinisation in the GSD population. C-banding in *L. whitii* revealed similar patterns to *C. ocellatus*, although we did not detect any sex specific heterochromatinisation between male and female *L. whitii* (Figure 5i–l). Multiple c-bands are observed in macrochromosomes and fewer in microchromosomes, and one macrochromosome pair and one microchromosome pair are highly heterochromatic (pairs 9 and 10; Figure 5i–l).

### 3.4. Telomere Repeats

**Figure 6:**
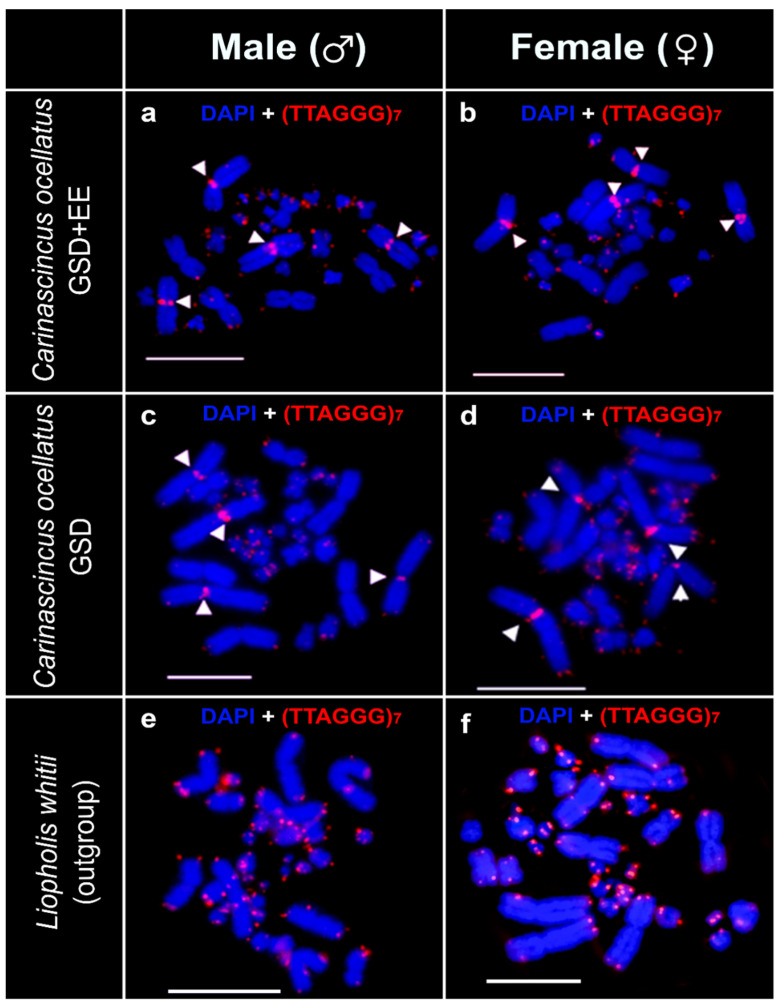
Chromosomal locations of the telomeric repeat (TTAGGG)_7_ sequence (red) in *Carinascincus ocellatus* GSD+EE (low elevation) male (**a**) and female (**b**), GSD (high elevation) *C. ocellatus* male (**c**) and female (**d**) and *Liopholis whitii* male (**e**) and female (**f**). White arrowhead indicates centromeric telomeres. Scale bars represent 10 µm.

Telomeric repeats were observed to hybridise onto the distal regions of both micro and macrochromosomes of males and females of all individuals (Figure 6a–f). Telomeric repeats are also observed at the centromeres of the two largest pairs of macrochromosomes in *C. ocellatus* (Figure 6a–d). We did not detect any sex specificity of any distal or interstitial telomeric sequences in either species (Figure 6a–f).

### 3.5. Microsatellite Motif (AGAT)_8_ Mapping

**Figure 7:**
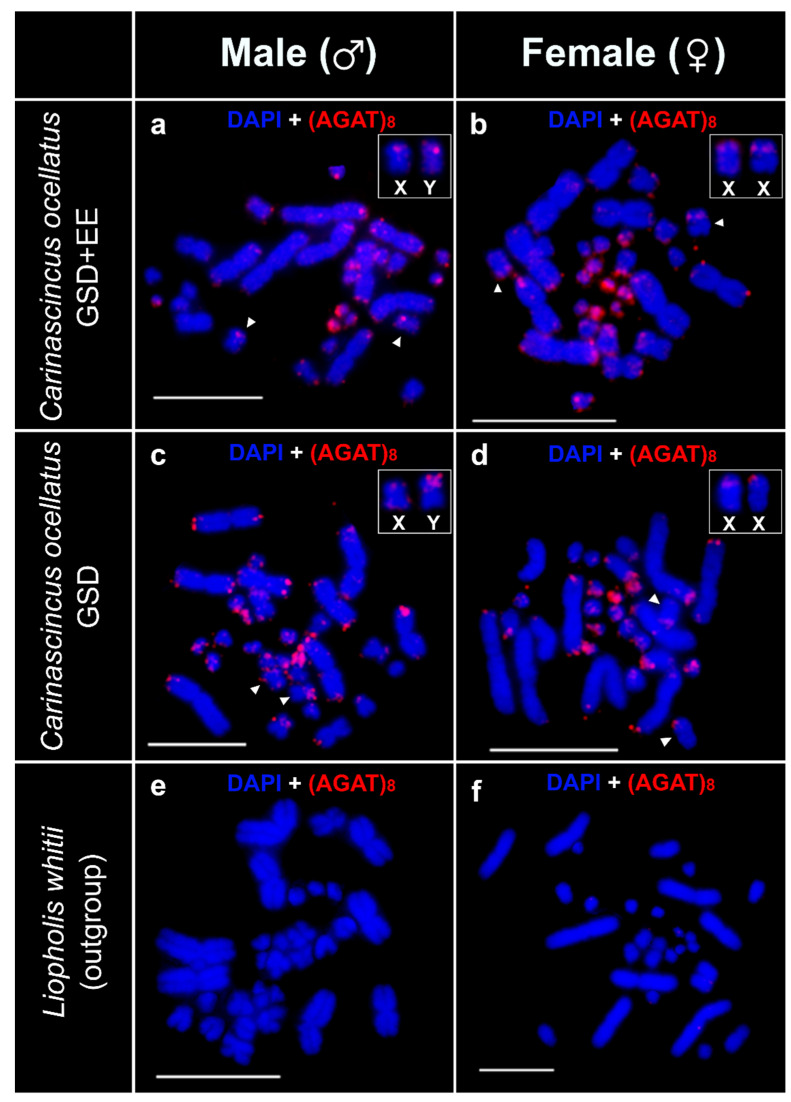
Chromosomal locations of the (AGAT)_8_ repeat (red) on *Carinascincus ocellatus* GSD+EE (low elevation) male (**a**) and female (**b**), GSD (high elevation) *C. ocellatus* male (**c**) and female (**d**) and *Liopholis whitii* male (**e**) and female (**f**). *C. ocellatus* sex chromosomes inset. White arrowhead indicates X and Y (the homologous pair) chromosomes in *C. ocellatus*. Scale bars represent 10 µm.

The (AGAT)_8_ probe hybridised onto telomeric regions of most of the macro and microchromosomes in males and females of both populations of *C. ocellatus* (Figure 7a–d). In addition, amplified hybridisation signals were observed in several microchromosomes in some males and females of both populations of *C. ocellatus* (Figure 7a–d). We also detected sex specific amplification of (AGAT)_8_ on chromosome pair seven of the GSD (high elevation) population of *C. ocellatus*. Hybridisation was observed at the distal ends of both arms of both members of this pair, however, in one member of the pair this signal was amplified on the p arm in males and identifies the Y chromosome (Figure 7c,d). This sex-specificity of the (AGAT)_8_ signal was not observed in the GSD+EE (low elevation) population. We did not detect any hybridisation signals of the (AGAT)_8_ probe in *L. whitii* (Figure 7e,f).

### 3.6. Sequential FISH of (AGAT)_8_ and Custom Y-Linked Probe Set

**Figure 8:**
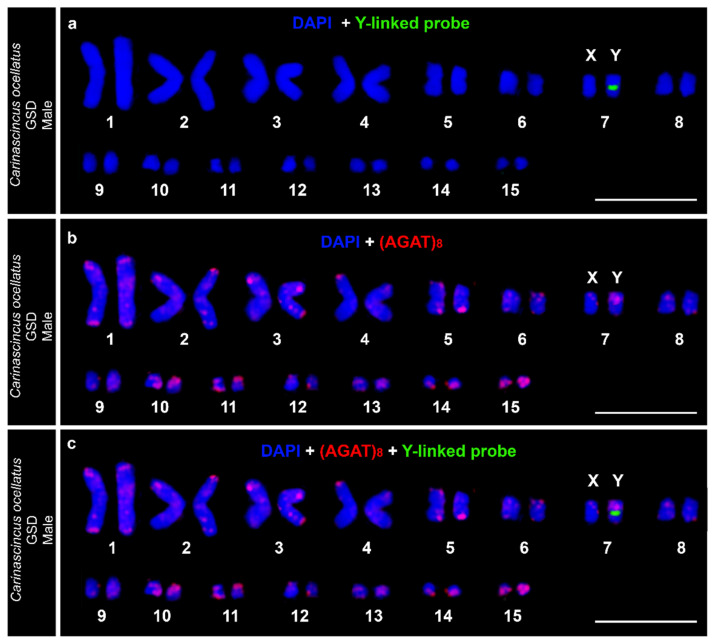
Sequential FISH and karyotyping in GSD (high elevation) population of *Carinascincus ocellatus* to confirm chromosome seven as the sex chromosome pair. (**a**) DAPI stained karyotype with FISH signals from Y-linked probe set (Pseudocoloured green) on *C. ocellatus* male; (**b**) DAPI stained karyotype with FISH signals from (AGAT)_8_ microsatellite probe (red) on the same metaphase; (**c**) Superimposed image of both layers indicates (AGAT)_8_ rich Y chromosome.

The identity of the X and Y chromosome pair in both GSD and GSD+EE populations of *C. ocellatus* was confirmed as macrochromosome pair seven (Figure 8) based on sequential FISH, which also confirmed population-specific (AGAT)_8_ signals on the Y chromosomes (Figure 8b,c, Figure S1).

### 3.7. Marker Homology

The only sequence with homology (e-value 2.34 × 10^−4^) to our *C. ocellatus* Y-linked probe set sequences that is relevant to sex determination or sex chromosome differentiation is a Sauria short interspersed nuclear element [67].

## 4. Discussion

*Carinascincus ocellatus* is a rare example of a species exhibiting population divergence in sex determination; GSD and GSD+EE occur in high and low elevation populations, respectively [51,52]. Here we also report divergence between the same populations in sex chromosome evolution. Given divergence between our *C. ocellatus* study populations occurred less than 1 million years ago [55,68], the mechanisms underpinning the transition in sex determination and those governing early sex chromosome evolution are potentially linked.

The high elevation, GSD Y chromosome is more heterochromatic and the p arm is AGAT rich compared to the low elevation, GSD+EE Y chromosome. This is consistent with observations of greater linkage disequilibrium of sex-linked DNA markers in the high elevation population [48], and that reduced recombination between the sex chromosomes is associated with repeat and heterochromatin accumulation on the Y chromosome during early differentiation from the X chromosome [19,20,69]. The differences in sex chromosomes with elevation can be explained by population-specific selection that drives the divergence in sex determination [52]. GSD is adaptive at high elevation because large interannual temperature fluctuations would produce maladaptive sex ratio skews if sex determination was thermosensitive [51]. Therefore, sex chromosomes with lower recombination around a sex determining locus are the result of selection against skewed sex ratios and thus selection towards balanced sex ratios and GSD. Selection for GSD+EE at low elevation occurs because of the sex-specific fitness benefits of climate-mediated birthdate variation in this population [52]. Considering the lower selective advantage of GSD at low elevation, there is less selection against recombination around the sex determining locus in this population, and hence lower divergence between X and Y. Population-specific repeat and heterochromatin accumulation (this study), and recombination between the X and Y chromosomes [48], therefore likely reflect differences in the size of the pseudoautosomal region (the region of the sex chromosomes that continues to recombine) of the Y chromosome in each population resulting from differential selection for GSD. One alternative is that population size differences have led to different rates of accumulation of mutations on the Y chromosome, however, the high and low elevation populations are of similar size [68] making this unlikely.

Observations of heterochromatin and AGAT repeat accumulation in the high elevation GSD population suggest that the ancestral sex chromosomes and sex determination was closer to the current situation in the low elevation GSD+EE population. An alternative hypothesis is that selection favouring recombination between sex chromosomes has driven the evolution of GSD+EE at low elevation from a GSD ancestor that possessed sex chromosomes similar to those in the high elevation population. However, this alternative hypothesis is unlikely because transitions from environmental to genetic sex determination in squamates are higher than the reverse, suggesting GSD is more stable [5]. Because both populations are characterised by a Y-specific region with significant homology to a class of retrotransposable elements implicated in recombination suppression [10,70,71], selection in the ancestral population, and indeed the low elevation population currently, may have favoured subtle variations in sex ratio with climate, rather than a strict TSD system. Population divergence in sex determination and sex chromosomes in *C. ocellatus* occurred without gene flow [68] and our results show that the accumulation of changes that accompany transitions can occur over short evolutionary time scales.

While divergence in *C. ocellatus* sex determination has arisen recently, it is super-imposed over a deep evolutionary conservation of sex-linked sequences. Comparisons against *Eulamprus heatwolei* (Scincidae) suggests conservation of Y chromosome sequences in *C. ocellatus* for ~79 million years (Figure 1; [35]). Likewise, heteromorphism of chromosome pair seven has been reported in two species of skink, *Bassiana duperreyi* [44] and *Pseudemoia entrecasteauxii* [69], both close relatives of *C. ocellatus* (Figure 1, divergence time [36,37] Mya, retrieved from TimeTree; [56,59]), suggesting a conserved role for pair seven as sex chromosomes (but requiring confirmation of sequence homology). The historical conservation of the sex-linked sequence, combined with the recency of transition in sex determination in *C. ocellatus*, is compatible with only a small number of genetic changes potentially underlying this transition [48].

Observations from *L. whitii* provide a contrasting perspective on sex chromosome evolution among Lygosomine skinks. Sex-specific chromosomes (Y or W) usually display increased heterochromatinisation [11,19]. Further, microsatellite and telomere repeat accumulation characterizes the sex chromosomes (Y and W) in a broad range of reptiles [57,58]. We found no sex-specific patterns of telomere accumulation in the karyotype of *C. ocellatus* or *L. whitii*. In addition, we found no evidence to suggest the AGAT repeat is involved in sex chromosome evolution in *L. whitii*. Our custom Y-linked probe set also lacked accumulation on any *L. whitii* chromosome. These absences suggest independent evolution of sex chromosomes in *L. whitii*. Independent evolution may include processes resulting in the loss of the Y chromosome region containing our *C. ocellatus* probe sequence and may also include accumulation of lineage-specific repeats. Sex chromosome evolution in subfamily Lygosominae may be associated with changes in chromosome number, because *C. ocellatus*, *B. duperreyi*, *P. entrecasteauxii* and *E. heatwolei* all possess a diploid chromosome compliment of 30, while *L. whitii*, belonging to a clade nested within these species’ clades, possesses 32 (Figure 1; [41,44,54,69]). Differences in the number of acrocentric chromosomes (*C. ocellatus* = 1; *L. whitii* = 2) suggest chromosome fission or fusion events during speciation and karyotype evolution [70,71]. Therefore, karyotype changes may favour the independent evolution of sex chromosomes and this may be one mechanism acting at the time of the split between the ancestor of *L. whitii* and other Lygosomine lineages with a diploid complement of 30. Sex chromosome origin coinciding with chromosome fission/fusion events is evident in Iguanids [72,73], and may represent a common mechanism throughout squamates. This can be confirmed in Scincidae via further experiments designed to identify sex chromosome homology and identity among closely and distantly related species, alongside karyotype analysis.

## 5. Conclusions

By examining the karyotypes of *C. ocellatus* with intraspecific variation in sex determination, we reveal that structural changes in sex chromosomes such as heterochromatinisation and repeat accumulation could be associated with such transitions. This is evidence of links between sex determination transitions and sex chromosome evolution. Through broader taxonomic comparisons, we also reveal a potential association between sex chromosome origin and karyotype evolution. Scincidae represents a valuable taxon for our understanding of diversity in sex determination, sex chromosomes and karyotype.

## Figures and Tables

**Table 1:** Number of male and female individuals and cells examined from whole blood culture of GSD (high elevation) and GSD+EE (low elevation) populations of *Carinascincus ocellatus* and a single population of *Liopholis whitii*. ‘Sequential’ FISH represents Y-linked and microsatellite FISH in series.

	GSD+EE *C. ocellatus*				GSD *C. ocellatus*				*L. whitii*			
	Male		Female		Male		Female		Male		Female	
	Ind.	Cells	Ind.	Cells	Ind.	Cells	Ind.	Cells	Ind.	Cells	Ind.	Cells
Karyotyping	3	73	3	50	3	129	3	64	3	43	3	67
c-banding	1	4	2	6	2	18	2	31	1	3	1	7
*FISH*												
Telomere	3	15	2	20	2	36	2	18	3	17	3	24
AGAT	2	28	2	14	2	43	2	28	2	14	2	24
Y-linked	3	19	2	16	2	33	3	18	3	12	3	19
Sequential	1	11			1	17						

## Data Availability

Data presented in this study are available in the article and supplementary material.

## Supplementary Materials

The following are available online at https://www.mdpi.com/2073-4409/10/2/291/s1, Figure S1: Sequential FISH in GSD+EE male, Table S1: Custom Y-linked probe set sequences.
