# Supplementary material for: Differences in Homomorphic Sex Chromosomes Are Associated with Population Divergence in Sex Determination in Carinascincus ocellatus (Scincidae: Lygosominae)
Source: Cells. 2021 Feb 1;10(2):291. doi: 10.3390/cells10020291 (PMC7912723; doi:10.3390/cells10020291)
Supplement: Supplementary file 1 [file cells-10-00291-s001.pdf]

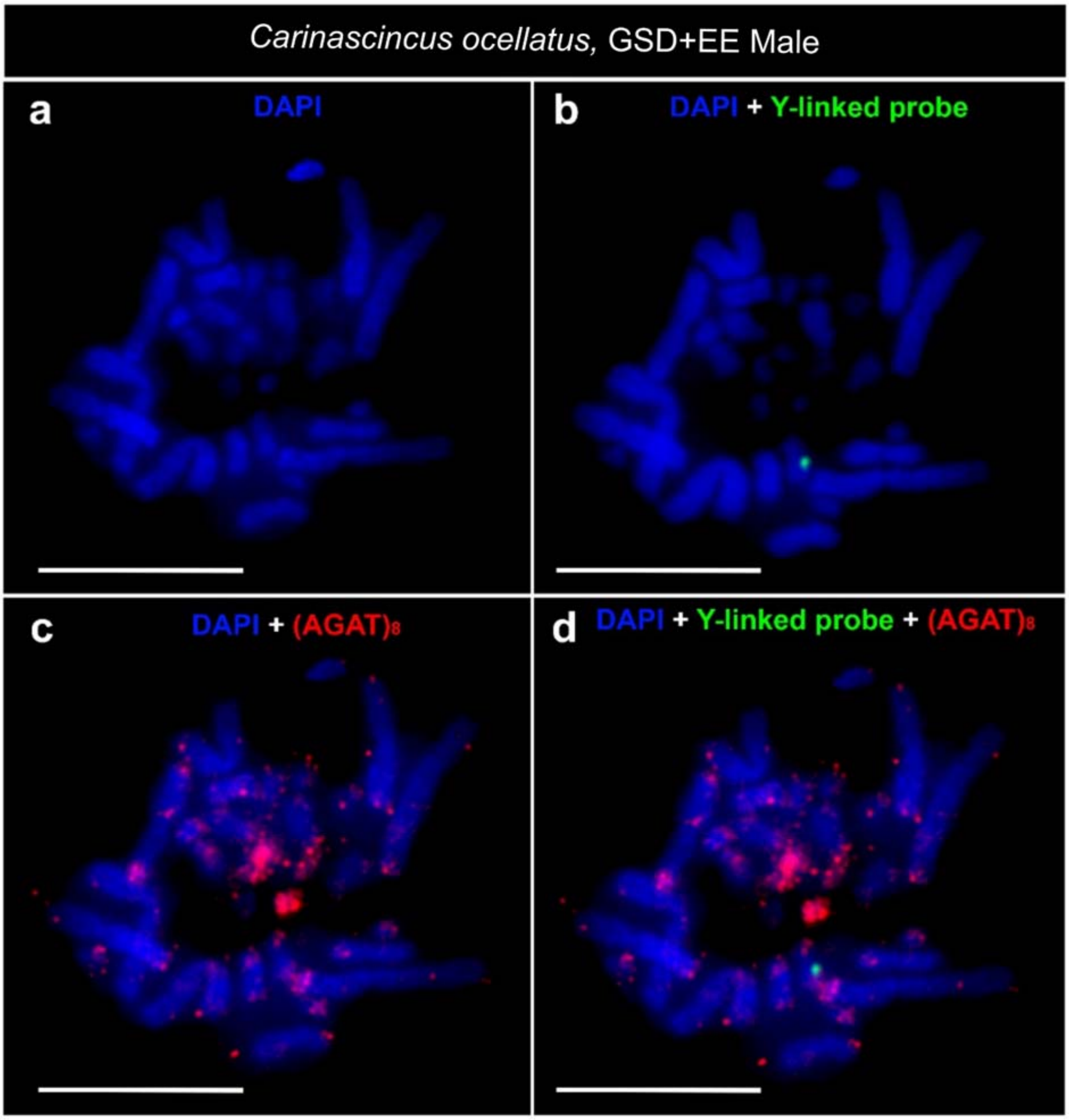

Figure S1: Sequential FISH with Y-linked probe set and (AGAT)8 probe in low elevation (GSD+EE) population of C. ocellatus. (**a**) DAPI stained metaphase spread of male C. ocellatus; (**b**) FISH with Y-linked probe set (Pseudocoloured image) on same metaphase; (**c**) FISH with (AGAT)8 microsatellite probe on same metaphase; (**d**) superimposed image of all layers (DAPI, signals from Y-linked probe set and signals from (AGAT)8).

Table 1. Custom Y-linked probe set sequences for *Carinascincus ocellatus* GSD and GSD+EE populations and homologs of our markers with publicly available sequences from vertebrates from NCBI BLAST [66].

Locus ID	Sequence	homology	E value
15694756	TGCAGTCATGTGGCCGGTATGCCACATATATGCCAAGGTGAACAAAACGCTG TTGCCTTCCAACCAAAG	*Elaphe dione* clone ela2 Squam-1 SINE repeat sequence.	2.34×10^{-4}
15694756	TGCAGTCATGTGGCCGGTATGCCACATATATGCCAAGGTGAACAAAACGCTG TTGCCTTCCAACCAAAG	*Azemiops feae* clone AFE-4 SINE, complete sequence	8.17×10^{-4}
15705269	TGCAGGACACTGGCAGGGCCAGAGGGTGCCCCAGCATCCCTCACCTGGG	NA	NA
15698539	TGCAGAAAAAGGGGTTGTGGCTATCAATTAAATTTTTACCTATATAAATTGCT TACCCACTTTG	NA	NA
15701508	TGCAGGTAAGTCTCACAGTGGGTGGCAGAGCTAGAGGGGGACACAGTAGAG AACGTTGGAGAGGGG	NA	NA
15727479	TGCAGCCGCTACAGGGAGACTGAGGGGGGATCATTTCCAGCCAGG	NA	NA
15704137	TGCAGGAAACTTATGTCAACTCTACAGGAAATGGAAGGCGAGACCACAGTT GATTTGG	NA	NA
15694656	TGCAGCTGTTACTGGTTGTCAGGGACTCCAGGCACCTCAGCTGTTTAGTGTGA TCTGAGGCAGGTCTC	NA	NA
15698519	TGCAGTTATGCAGGAAGCAGCCATTCCTGTGTCTGGCTCTTGGTTCACCAGGC CCACTTTGTCTGCACT	NA	NA
15704111	TGCAGCATTTCTGAGCCCAGCTCTGGGCGTGCGCACTGGGGACAATGAACGC GCCAGTGCTGAGTGCA	NA	NA
15712147	TGCAGCAAAAGCCTCAAACTTGTGCTGGATATAGCGCAAGCGG	NA	NA
15694951	TGCAGGCTGCTCTGGAAATGGCTCTTCTCGGTGTGCAGAGCTCCTGTG	NA	NA
15706148	TGCAGAAAGGGTGAAAACGTTCCTTTATCCATTCAGTG	NA	NA
15694660	TGCAGCTTCCTTCTCCCCATTGCTGAAGATCAAGGGATGCTCTGTAGCATCAT CCCATGCAGCACAGAG	NA	NA
15694518	TGCAGCTTCAAATCTCCAGGCCACTTGGCCTAGTTTTTTTAACAGCTTCCAGC TGCATCTAAAAGGGGA	NA	NA
15694646	TGCAGCTCAAGAACTCTGCCCAGTTTTCCCCCTTAAACAGTGCGGTCTGAGC ATAGTTTCCCCTTTTAG	NA	NA
15694818	TGCAGTTTTGGACTGTTGCGAGGAGGGGTTTACAATAGTGGAAGAAACTTCT GCTATTGTAAATTGGCT	NA	NA
15700798	TGCAGGACTTAAAAGCTGCATATGAAGTGTGCATCTTAGGAAGTAAATTTTG CTGAACCTCAGCAGGG	NA	NA
15704112	TGCAGCCAAGGCTGTAGCCATGAGGTGGGGGAGGGACTTCGTCCTAGTCCCG CCTCCATGTAGATTCC	NA	NA
15725981	TGCAGGGAGGGAACAGGGATTGGGCTGCAATCCTGTTGTAAGAGAGCACTA TGAACACAATCCTAAG	NA	NA
15694991	TGCAGTACTTTCCTGGAAAGAAGCCCTATGTCTAAGTGCATAGTG	NA	NA

15694717	TGCAGGGTTTGGCTCTACTGGGGTGAATGGCAAGTGGGGCTCTAGAGTGATGAAAGGAAGTTCTTTGTA	NA	NA
15694757	TGCAGTCCAGATGAAAGCTGGGCAGGCAATGGAAGAGCCTTATCCTCTAGGCCAGGTG	NA	NA
15694779	TGCAGTGACCAGCAGTGGCGTAGCTAGATGAGGGTGCAAAGCACTGTTTTGCAGGAAACTTCACCGCAG	NA	NA
15694858	TGCAGATCTAAAACACCTAACCTTTAAGACGGTCTTTCTAATTTCAGCCACATCGG	NA	NA
15694872	TGCAGCAATCTAGGAACTCTGTTTAAGAACGATTGGCAGCGTG	NA	NA
15694881	TGCAGCAGCCATTGTAATTTGAATGTGCGCCGTCCGGATTACATAGCGCGCATG	NA	NA
15694946	TGCAGGCCTCCCAGGATCTGGAGCAGGCATACTAGCAAGAGGAATTTCTGCCGG	NA	NA
15698495	TGCAGGTATATCTCATTAAAAATGCCAGCCCATAATATTGATTGTATGCCTGTCAGTCAAATGAGTAAG	NA	NA
15701619	TGCAGGTGCACGAAGTCCTTTGGGGAGAGTCATTCATTAGTATTGAATGACTGGATTGACCTTCCACA	NA	NA
15704127	TGCAGCGTGTCAACCCCCTCTGGCGCGTCACCCTCACCCGTGCCAGTGGAGAGGACTGCTCCTCAACTG	NA	NA
15717728	TGCAGGGCTTGGGCTGCAATCCTGTTGTAAGAGAGCACTATGAACACAATCCTAAG	NA	NA
